# The Involvement of *NRF2* in Lung Cancer

**DOI:** 10.1155/2013/746432

**Published:** 2013-03-18

**Authors:** Alison K. Bauer, Thomas Hill, Carla-Maria Alexander

**Affiliations:** Department of Environmental and Occupational Health, University of Colorado Anschutz Medical Campus, Mailstop B119-V20, Room. 3125, 12850 East Montview Boulevard, Aurora, CO 80045, USA

## Abstract

Nuclear factor, erythroid-derived 2, like 2 (*NRF2*) is a key regulator of antioxidants and cellular stress responses. The role of *NRF2* in pulmonary neoplasia, a diverse disease for which few biomarkers exist, is complicated and appears to depend on several main factors including the existence of activating mutations in *NRF2* and/or loss of function mutations in *KEAP1* and the stage of carcinogenesis studied, particularly in the mouse models tested. Therapeutic strategies for lung cancer targeting *NRF2* have observed mixed results, both anti- and protumorigenic effects; however, these differences seem to reflect the mutation status of *NRF2* or *KEAP1*. In this paper, we will discuss the studies on human *NRF2* and the mechanisms proposed, several mouse models using various mice deficient in *NRF2*, as well as xenograft models, and the chemotherapeutic strategies using the *NRF2* pathway.

## 1. Introduction

Lung cancer mortality rates are the highest among all cancers worldwide [1, 2]. Although smoking rates have decreased in the US, many countries have observed few changes in smoking habits, and 10–20% of lung cancer patients are nonsmokers [1, 3, 4]. Thus, understanding and identifying novel pathways for therapeutic targets is a primary goal in research on pulmonary neoplasms. Non-small-cell lung carcinoma (NSCLC) has the highest incidence rates and most studies focus on its specific subtypes, squamous cell carcinoma or adenocarcinoma (AC), although there are several other subtypes under the NSCLC heading [2]. NSCLC is also the most common among smokers as well as the only lung cancer found in nonsmokers [5]. NSCLC develops in the central bronchi in squamous cell carcinoma (SCC) and in the bronchioles and alveoli in adenocarcinoma (AC). Small-cell lung carcinoma (SCLC) accounts for ~20% of lung cancer and is almost exclusively associated with a smoking etiology [5]. SCLC tumors are centrally located in bronchi and express neuroendocrine markers [5]. While some lung tumor subtypes, such as SCC, have early precursor lesions, most have few early biomarkers for detection [5]. We refer the readers to reviews on lung cancer for more on the etiology [5–7].

This paper and special issue of this journal will focus on a molecule called nuclear factor, erythroid-derived 2, like 2 (NRF2), a master transcription factor that regulates antioxidant response element- (ARE-) mediated expression of antioxidant enzymes and cytoprotective proteins [8]. Oxygen is essential for the survival of all aerobic organisms and its metabolism results in partially reduced oxygen byproducts collectively known as reactive oxygen species (ROS) [9, 10]. Excess ROS causes oxidative damage to cellular DNA, lipids, and proteins; genetic changes and/or epigenetic alterations can lead to the dysregulation of oncogenes and tumor suppressor genes, ultimately contributing to the pathogenesis of cancer [11, 12]. To alleviate this oxidative stress, there are several antioxidative stress responses, many regulated by NRF2. NRF2 expression is abundant in tissues where detoxification reactions occur, including the lung [9], and under normal physiological conditions it interacts with its own negative regulator, Kelch-like ECH-associated protein 1 (KEAP1) [13]. KEAP1 is a cytoplasmic, cysteine-rich, actin-bound protein that sequesters NRF2 in the cytoplasm and directs it to CUL3 E3 ligase for ubiquitylation and subsequent degradation by the proteasome [9, 10, 13]. In times of oxidative stress, selected KEAP1 cysteines become oxidized leading to a disruption of the KEAP1-NRF2 complex and the release of the NRF2 peptide. NRF2 then translocates to the nucleus to transcribe genes encoding various antioxidant proteins and metabolic enzymes collectively known as phase II detoxifying enzymes [10]. Alternative pathways for NRF2 activation are through the phosphorylation of NRF2 by protein kinase C (PKC) or RNA-dependent protein kinase R- (PKR-) like endoplasmic reticulum kinase (PERK), resulting in the release of NRF2 from KEAP1 [14–16].

The role of NRF2 and KEAP1 in cancer development has been highly controversial and has led to many theories including NRF2 as an oncogene, or its manipulation by an oncogene, specifically in the lung [13, 17–20]. It is clear that the findings in lung cancer differ from those observed in most other organ systems, or even other pulmonary diseases, such as emphysema [21], hyperoxia [22], and respiratory syncytial virus [23], where disease symptoms significantly worsen in the absence of NRF2. Thus, the mechanisms driving these tumorigenic responses appear unique to tumor development. However, some studies examining activating mutations in *NRF2* or loss of function of *KEAP1* in human cancers, such as esophagus, skin, and ovarian cancers, did find one or more of these mutations altering the NRF2 pathway, which suggests protumorigenic involvement [24, 25] in these extrapulmonary tissues. We will only discuss NRF2 in the context of lung cancer in this paper, but many other mouse models, including colon, bladder, liver, and mammary, have demonstrated that a lack of NRF2 increases the potential for carcinogenesis [26–29]; this varies greatly in pulmonary neoplasias depending on the model tested. We will first discuss the human studies that have been done including the polymorphisms identified and their proposed effects, mouse models for lung cancer and NRF2, and the chemotherapeutic targets that use NRF2 in either a protumorigenic or antitumorigenic manner in lung. 

## 2. Human *NRF2* Studies

The lung is an organ of high surface area that is intimately associated with the central compartment to facilitate gas diffusion. Therefore, it is a seminal point of exposure to environmental toxicants such as cigarette smoke, ozone, particulates, and exhaust emissions such as polyaromatic hydrocarbons and peroxyacetyl nitrate [30]. Such toxicants have been implicated in the incidence of lung cancer and linked to increased burden of ROS in human tissues [31], as well as the upregulation of antioxidant-selective genes [32]. The growing tumor and its microenvironment are an additional source of ROS from accelerated mitochondrial function required for rapid cell growth and division in the proliferative phase [33]. Activating or stabilizing modifications of NRF2 increase its nuclear translocation in response to hypoxia or ROS in A549 cells (AC cells), and it has been suggested that this is a cell survival mechanism [34, 35]. In addition, analyses of tumor tissue from a variety of cancers, including lung, display overexpression of the phase II antioxidant enzymes regulated by NRF2, such as glutathione-S-transferase (GST) and NADP(H): quinone oxidoreductase 1 (NQO1), which are both known to facilitate the elimination of reactive, oxidized metabolites [30, 36]. While studies indicate that there is ample evidence to support the involvement of NRF2 in cancer biology [37–40], the dominant focus in human research are somatic mutations in *NRF2* and/or its repressor protein KEAP1, that confer either enhanced tumor escape from apoptosis or resistance to a variety of cancer chemotherapeutics [41]. Interestingly, gain of function mutations in *NRF2* are more closely associated with chronic smoking while loss of function *KEAP1* mutations are not [40, 42, 43]. For further discussion on cigarette smoking and NRF2, we refer the readers to Muller and Hengstermann, 2012 [42].

### 2.1. Studies Examining the Effects of *NRF2* Mutations

One group of somatic mutations identified specifically in AC and SCC lung tumors occurs within the Neh2 domain of the *NRF2* gene [43]. This domain contains a bZIP region fused to a cap n' collar (CNC) region and governs both the ability of NRF2 to dimerize with MAF proteins as well as DNA binding to ARE regions in the target genes [44, 45]. Of particular significance are mutations within the DLG (amino acid 27–32) and ETGE (amino acid 77–82) regions of the domain, as they affect the binding affinity to the KELCH domain of KEAP1 and inhibit redox-sensitive repression by KEAP1 that normally controls the basal levels of NRF2 expression [41, 46]. The inability to maintain NRF2 protein expression at or below a basal level has been directly linked to poor prognosis in clinical patients diagnosed with either lung AC or SCC [40, 43, 47]. Investigations in human cancer cell lines have linked elevated NRF2 expression with resistance to specific anticancer chemotherapeutics [48–50]. An *in vitro* study of the human cancer cell lines A549, NCI-H292 (mucoepidermoid cells), and RERF-LC-Ai (SCC cells) (ranked from highest to lowest constitutive expression of NRF2) demonstrated that resistance to cisplatin was proportional to NRF2 expression [51]. Expressions of antioxidant enzymes, phase II metabolic enzymes, and drug efflux pumps in these cell lines were also elevated in proportion to NRF2 and sensitive to siRNA knockdown of *NRF2*. The *NRF2* siRNA knockdown also profoundly inhibited the cellular proliferation of the A549 cells. Similar findings were observed in a study of human carbonyl reductase 3 (CBR3) and its regulation by NRF2 [52]. This study utilized human AC cell line A549, SW-480 (colon), HT-29 (colon), and the hepatocellular HepG2 cell line. In all lines, the magnitude of NRF2 expression reflected the degree of CBR3 induction/expression, but siRNA knockdown of *NRF2* in A549 cells reduced the levels of CBR3 to just 30% of control. Multidrug resistance protein 3 (MDR3), which has been linked to drug resistance in NSCLC, is also known to be directly induced by NRF2 in both NSCLC tumor tissue and immortalized ATCC cell lines (DU-145 prostate; H1666, H1650, and A549 (AC); H358-unspecified NSCLC) [53]. The end result is that NRF2 levels alone have become a prognostication factor for patient treatment decisions in non-small-cell lung cancer [54, 55].

### 2.2. Studies on the Effects Elicited from Alterations in KEAP1

 Due to the presence of six KELCH regions in the binding domain of NRF2, a loss of NRF2 inhibition by KEAP1 should also occur as a result of mutations in the KELCH binding region of the KEAP1 protein itself. A study of Japanese lung cancer patients (AC, SCC, large cell carcinomas (LCCs), and SCLC) documented these *KEAP1* mutations and identified them as a source of constitutive expression of MDR proteins, phase II enzymes as well as specific cisplatin resistance in cultured lung AC cells [56]. Genetic studies of human lung tumors (AC, SCC) further substantiate this, and some suggest that tumor types may have distinct patterns of *KEAP1* mutation frequency [57, 58]. In a study meant to further describe the translocation of NRF2 from KEAP1 to the nucleus, it was found that the NSCLC cell lines A549 and H460 (LCC) (both *KEAP1* mutants) had constitutively high MDR3 levels. The authors hypothesized that the induction of MDR3 might be purely due to increased nuclear translocation of unbound NRF2 independent of the *KEAP1* mutation. However, the levels of MDR3 in these cell lines failed to superinduce when treated with 4-hydroxy-2-nonenal, which was shown to enhance the nuclear translocation of NRF2 in the human bronchial epithelial cell line 1 (HBE1) and in a NSCLC cell line H358 (bronchoalveolar carcinoma), which lack the *KEAP1* mutation [59]. The cytoprotective benefits of these multi-drug resistance mechanisms are mixed. A recent study in H358 demonstrated an aryl hydrocarbon receptor (AhR): NRF2 coinduction of multiresistance protein 4 (MRP4) actually lowers DNA-adduct formation in cells exposed to either benzo[a]pyrene (B(a)P) or 2,3,7,8-tetrachlorodibenzo-p-dioxin (TCDD) via cellular efflux of the inactive metabolites [60]. However, the same combination of AhR and NRF2 is enhanced by the exposure of lung cancer cells to cigarette smoke condensate, triggering upregulation of the xenobiotic pump ABCG2 and resistance to chemotherapeutics [61, 62]. Thus, enhanced expression of MDR proteins through NRF2 can elicit both pro- and antitumorigenic responses.

 While the nature of *NRF2 *mutations involves direct DNA substitutions that result in a loss of binding affinity to the KELCH region, the *KEAP1* mutations create gross structural alterations and stearic hindrance to the formation of the NRF2 : KEAP1 complex. In addition, *KEAP1* mutations have the potential for epigenetic inactivation. Methylation of the *KEAP1* promoter region was observed with a frequency of up to 47% in tissues from NSCLC patients (both AC and SCC), with somatic mutations in only 15% and loss of heterozygosity in 21%—no methylation was detected in normal controls [63]. Similar epigenetic findings have been reported for a prostate cancer cell line (DU-145) and associated with resistance to a variety of chemotherapeutics and radiation—acquired traits that are reversed with siRNA silencing of *NRF2* [64]. 

### 2.3. Downstream Mechanisms

While the *KEAP1* mutation often increases ARE gene targets indirectly by increasing the availability of NRF2, it has also been shown to directly amplify induction of PPAR*γ* and confer chemoresistance [65]. *KEAP1* mutations can also directly interfere with BCL2 degradation to enhance cellular escape from apoptosis [66]. Thus, the *KEAP1* mutation itself can offer a significant survival adaptation to cancerous and precancerous cells. The combination of the *NRF2* and *KEAP1* mutations is particularly advantageous. For example, in studies in A549 cells with a mutant *KEAP1* insertion, not only does the *KEAP1* mutation interfere with BCL2 degradation, but loss of NRF2: KEAP1 dimerization leads to direct induction of BCL2 by NRF2, increasing the amount of available BCL2 for apoptotic escape [67]. These cells displayed increased resistance to etoposides and either UV or gamma irradiation, characteristics that were lost by siRNA knockdown of either *NRF2* or* BCL2*.

 The current state of knowledge regarding NRF2 and KEAP1 in human lung cancer suggests that both apoptotic escape and resistance to anticancer treatments are responsible for the poor prognosis associated with elevated NRF2 levels and KEAP1 dysfunction. Interestingly, if one examines the frequency of common mutations in human lung tumor tissue, for both SCLC and NSCLC (*p53*, *RB*, *BCL2*, etc.), these are found to occur at a rate exceeding 50% in both types [68]. A genomic study of SCC reports a *NRF2/KEAP1* mutation frequency of 34% in 178 tumors from affected individuals [69]. A recent review further calculates the frequency of *KEAP1* and *NRF2* mutations for SCLC and NSCLC samples at approximately 25% overall and proposes that the acquisition of ARE-driven enhancement of tumor survival is likely to be the result of an insult that drives tumor promotion or progression rather than the initiation phase [41]. 

## 3. Animal Studies Assessing the Role of *NRF2* in Lung Carcinogenesis

 Animal studies on the NRF2 pathway have used *Nrf2*-deficient mice on multiple backgrounds, *Keap1*-deficient mice, xenograft models, metastasis models, as well as *Nrf2*-deficient mice crossed with a *K*-*Ras* ^G12D^ mouse. We will discuss all of these models herein. The tumorigenic potential of human activating *NRF2* mutations in an altered HEK293 cell line expressing mutant *NRF2* (T80R and L30F, gain-of-function mutations in cancer) [43] was examined and found to induce tumorigenesis *in vivo *using xenografts in immunodeficient mice [70]. The xenografts consisted of mutant *NRF2*-induced tumors that were poorly differentiated with many microvessels, NQO1 production (downstream of NRF2), and an occasional metastasis to the liver [70]. Additionally, the mutated *NRF2* was dependent on the mTOR pathway, identified using mTOR inhibitors. Certain heterozygous mutations in *KEAP1*, previously identified in human lung tumors, were also found to have a dominant negative effect on wild-type *KEAP1*, using an *in vivo* system in transgenic mice [71]. Specifically, *KEAP*1^G430C^ or *KEAP*1^G364C^ coexpressed with the WT *KEAP1* in mice (mixed background, 129Sv/J, C57BL/6J, and ICR) resulted in significant hyperactivation of genes downstream of NRF2, such as *Nqo1*, supporting a dominant-negative effect of mutant *KEAP1* [71]. Thus, while these studies are not lung-specific, they demonstrate that *NRF2* and *KEAP1* mutations identified in human lung cancer have functional effects in animal models.

In a primary lung cancer model using urethane, a well-established model [72, 73] to induce tumors, *Nrf2*-deficient mice (^−/−^) were significantly less susceptible to tumor development than the *Nrf2* ^+/+^ mice (WT; BALB background strain) [37]. However, *Nrf2* ^−/−^ mice had increased hyperpermeability, inflammatory cell infiltrates, including monocytes, macrophages, and lymphoctyes, and elevated myeloperoxidase, that was suggestive of increased numbers of PMNs, compared to the *Nrf2* ^+/+^ mice 11 wks following urethane. Significant reductions in the early adenomatous lesions in the *Nrf2* ^−/−^ mice were also observed 12 wks following urethane with concomitant increases in apoptotic cells, compared to the wild-type mice [37]. Thus, the cell death pathways involved in apoptosis and necrosis, such as significant increases in LDH in the *Nrf2* ^−/−^ mice compared to *Nrf2* ^+/+^ mice, support the hypothesis that the urethane-initiated epithelial cells, such as the type II alveolar pneumocyte or bronchiolar Clara cell, both progenitor cells for lung AC [74], were more susceptible to cell death in the mice lacking Nrf2. The *Nrf2* ^+/+^ mice, therefore, have both a growth advantage and increased cytoprotection for tumorigenesis.

 A transcriptome study was also performed to determine the differences between strains (*Nrf2* ^−/−^ and *Nrf2* ^+/+^) involved in these responses to urethane at an early and late time point. At the 12 wk time point, Nrf*2-*modulated genes involved glutathione metabolism, cell-cell signaling, oxidative stress, and immune responses. In the more advanced stage, the Nrf2-dependent genes associated with cell cycle/proliferation and cell death, correlating in direction and magnitude with the increased death of initiated cells in the *Nrf2* ^−/−^ mice. At 22 wks, PMNs were also significantly increased in the tumor-bearing lungs of *Nrf2* ^+/+^ mice compared to *Nrf2* ^−/−^ mice, as well as the chemokine *Cxcl1* (*Kc*) [37]. Altogether, these studies demonstrate that in a primary mouse lung cancer model, NRF2 promotes survival properties and supports the human studies demonstrating resistance to anticancer drugs as well as increased malignancy. Interestingly, when an additional primary tumor model (MCA/BHT) was used with these strains, no differences were observed, suggesting NRF2 protection may be both carcinogen and stage dependent [37]. In addition, because urethane is not considered a mimetic of cigarette smoke, although it is a component of cigarettes [75], the underlying mechanisms of lung cancer may be different.

 As described earlier, ROS are often involved in initiating disease states (i.e., cancer), and thus the concept that reduction of ROS may lead to increased carcinogenesis is against the normal doctrine [76]. However, studies demonstrated that when the oncogenes *K*-*Ras* ^G12D^or *B*-*Raf* ^V619E^ (mutated, activated) were expressed *in vitro* in murine NIH3T3 or mouse embryonic fibroblasts (MEFs), ROS levels as measured by 7,8-dihydro-8-oxo-2′-deoxyguanosine (8-oxo-dGuo), and free hydrogen peroxide or superoxide, were all subsequently decreased [77]. In contrast, Nrf2 activity and its downstream genes were elevated and the ratio of reduced-to-oxidized glutathione increased (GSH/GSSH) [77]. These oncogenic pathways (K-Ras and B-Raf) were found to signal through MEK, ERK MAP Kinase, and the AP-1 transcription factors, Jun and Fra1, finally inducing Nrf2, which led to the antioxidant responses observed [77]. These *in vitro* findings were then validated in several *in vivo* mice models, including those for lung and pancreatic cancer. *Nrf2* ^−/−^ and *Nrf2* ^+/+^ mice were bred to the *K*-*Ras* ^G12D^ mice (B6/129/SJL background strain) in lung tumorigenesis studies which demonstrated that the *Nrf2* ^−/−^ mouse had a significant reduction in *K*-*Ras* ^G12D^-initiated lung tumors compared to WT mice [77]. These mice also had reduced Ki67 staining indicating less proliferative activity as well as overall decreases in adenomas, adenomatous alveolar hyperplasias and bronchiolar hyperplasias [77]. The pancreatic cancer model also demonstrated that in the absence of Nrf2, pancreatic intraepithelial neoplasia was significantly reduced [77]. Thus, it appears that Nrf2 can be regulated by specific oncogenes (K-Ras, B-Raf) to increase tumorigenesis by reduction of ROS through detoxification and antioxidant responses that creates a more favorable cellular microenvironment. The findings described here support the other primary mouse study which suggests that the initiated cells are reduced in mice lacking *Nrf2* and therefore, the cellular environment in those mice lacks the favorable protections of mice with sufficient *Nrf2* [37]. 

Lastly, in a Lewis lung carcinoma (3LL) mouse metastasis model, *Nrf2*-deficient mice on a C57/BL6/J background developed a significantly higher number of lung metastatic nodules than wild-type mice [78]. The total cancer incidence in the *Nrf2*-deficient mice was 100% compared to 28.6% in the WT mice. Pulmonary and bone marrow inflammation, including myeloid-derived suppressor cells (MDSCs), was also elevated in the *Nrf2*-deficient mice bearing metastatic nodules. MDSCs can suppress CD8^+^ T-cell populations through reactive oxygen species (ROS) and thus may be suppressing the immune response in these animals [79]. *Keap1* mutant mice with increased levels of Nrf2 protein were resistant to metastasis [78] and demonstrated reduced levels of ROS. In these studies, Nrf2 appears to play a role in the prevention of metastasis, which is the reverse of findings observed in other mouse lung cancer studies. However, the mechanisms regulating metastasis differ from the earlier stages of carcinogenesis, which were modeled in the other mouse studies. Additionally, the strain background used in the metastatic studies (B6) differs from those used in the previously described mouse studies (BALB and B6/129/SJL). These strains are known to differ in many phenotypes, including the polarity of their immune systems, which could influence responsiveness [80].

 As of yet, no studies in mice have examined the effects of altered Nrf2 in SCLC or SCC.

## 4. Chemotherapeutic Strategies Using the *NRF2* Pathway

By the nature of its cellular functions of alleviating oxidative stress, NRF2 is important in the prevention of disease onset and progression and has established a definitive role in cancer prevention [10, 12, 13, 18]. As mentioned above, using *Nrf2*-knockout mice in a LLC model demonstrated that the absence of *Nrf2* led to accelerated colonization and proliferation of metastatic cancer cells in the lungs [78]. However, recent genetic evidence demonstrates an upregulation of NRF2 in various human cancers including lung cancer, thereby suggesting possible protumorigenic involvement in several stages of cancer, such as promotion and progression. Along with increased NRF2 signaling, mutations in *KEAP1* and *NRF2* and constitutive expression of NRF2, are known to enable tumor cells to hijack the NRF2 pathway as a mechanism to resist chemotherapeutic agents [10, 43, 56, 58, 81, 82]. Cancer chemoprevention/chemotherapy is the use of plant-based (phytochemicals) or synthetic chemical compounds to prevent, suppress, delay, or reverse the development of invasive cancer. This concept has been expanded to target all stages of cancer development [83, 84]. One strategy includes the blockade of DNA adducts by reducing the formation of reactive carcinogenic species and stimulating their detoxification via modulation of phases I and II enzymes [84]. The premise that many chemopreventive drugs mediate their beneficial effects against carcinogenesis via the NRF2 pathway, contrasted with the idea that NRF2 may be prooncogenic or enhance chemotherapeutic resistance has caused controversy in the literature [13]. It is, therefore, important to review the context in which various therapies involve the NRF2 pathway to determine when it would be advantageous to use drugs to stimulate or to inhibit NRF2 in lung. We refer the readers to the following excellent reviews for NRF2 and general chemoprevention [12, 82], while we focus on lung herein. 

### 4.1. Studies Demonstrating Beneficial Effects of Stimulating *NRF2*

Dietary and medicinal plants are major sources of phytochemicals, which have played an important role in cancer treatment [83]. Dietary components that increase reduced glutathione (GSH) levels and induce phase II enzymes are known to activate transcription through the NRF2-ARE pathway. While various research studies have shown that human consumption of cruciferous vegetables, such as broccoli and brussel sprouts, both induces NRF2 and decreases risk of lung cancer [85–89], it should be noted that further chronic studies need to be implemented to confirm this linkage. Because polymeric black tea polyphenols (PBPs) protect against B(a)P-induced DNA adduct formation *in vitro*, Patel et al. investigated the role of Nrf2 in phase II enzyme induction by PBP extract in murine pulmonary tissues *in vivo *[84]. Pretreatment with PBP followed by sacrifice 1 day post-B(a)P induced total cellular levels of Nrf2 protein and increased nuclear accumulation of Nrf2 in the lungs. PBP extract also induced ARE-mediated *Nqo1* and *Gst* gene expressions, while the *Keap1* levels in the lungs remain unaltered. These results suggest that homeostatic maintenance of KEAP1 levels and the presence of phytochemicals at the beginning of initiation may be linked to a possible role of NRF2 acting as a tumor suppressor. Some phytochemicals utilize other pathways in conjunction with the NRF2 pathway to affect their anticancer activities. Curcumin, the principal curcuminoid of the Indian spice turmeric, has exhibited anti-initiating effects via the transcriptional regulators of phase I and II enzymes in mice [90]. Previous to inoculation with B(a)P as the carcinogen, mice were treated with dietary curcumin and the mechanisms of curcumin-mediated anti-initiation were investigated. Curcumin inhibited B(a)P-induced phase I enzyme activities by significantly decreasing AhR-DNA binding and thereby decreasing the subsequent activation of phase I enzymes. Curcumin also enhanced nuclear translocation of Nrf2 and Nrf2-ARE binding *in vivo*, leading to increases in phase II enzymes in the lungs [90]. Recent studies (described earlier) using B(a)P and dioxin demonstrated coinduction of AhR and NRF2 leading to a reduction in DNA-adduct formation and also supporting the curcumin findings [60]. However, due to the anti-inflammatory effects of curcumin [91] and the known importance of inflammation in lung cancer [92–94], involvement of other pathways cannot be ignored.

 While the previously mentioned studies involve pretreatment with chemopreventive compounds, there is also evidence that Nrf2 has a postinitiation role in experimentally induced lung carcinogenesis. Sulforaphane (SFN), an isothiocyanate isolated from cruciferous vegetables such as broccoli, is the most potent naturally occurring inducer of phase II enzymes, and although most pathways induced are NRF2-dependent, some NRF2 independent mechanisms have been shown, such as direct regulation of glutathione levels [95]. One *in vivo* investigation determined if Nrf2 contributes to pulmonary protection based on the timing of treatment with the phytochemical [96]. Whether SFN was introduced prior to the first dose of the carcinogen or after cellular initiation, increased phase II enzymes, decreased phase I enzymes and significant reduction of oxidative damage were observed [96]. Collectively, the NRF2 pathway appears important in facilitating chemopreventive measures in the lungs, as long as the pathway itself has not been altered/mutated. 

 There have also been some recent breakthroughs for new therapies, most notably in regards to suicide gene therapy and pro-drug activation of tumor-selective compounds [97, 98]. A lentiviral (LV) vector expressing herpes simplex virus thymidine kinase (HSV-TK/GCV) under the regulation of ARE (LV-ARE-TK/GCV), was constructed and its constitutive ARE hyperactivity was used to selectively target lung cancer cells for suicide gene therapy [97]. The vector was tested in human lung AC cells and a mouse xenograft model of lung cancer. In both settings, the vector was effective in decreasing cell viability and tumor size [97]. A drug in phase II clinical trials designed to promote tumor hypoxia was recently found to be bioactivated by a novel nitroreductase (AKR1C3) that was directly controlled by NRF2 levels. Subsequent microarray analysis of 2490 cancer patients demonstrated normative upregulation of AKR1C3 in tumor tissues, suggesting that NRF2 elevation and its sequelae could be used to enhance the specificity and efficacy of novel chemotherapeutics [98].

### 4.2. Studies on the Effects of Inhibition of the *NRF2* Pathway

Yamamoto and colleagues found a high incidence/frequency occurrence of loss of KEAP1 function in patients with lung cancer [9, 56, 99]. The low KEAP1 activity resulted in multiple effects and ultimately resistance to chemotherapeutic agents, for example, cisplatin [9, 56, 58]. Thus, there is a strong rationale for the development of new NRF2 inhibitory agents for the use in cancers in which genetic mutations cause constitutive activation of the NRF2 pathway [13]. In addition, NRF2 expression in A549 and H460 cells has also been shown to be protective against toxicity of ionizing radiation and may result in tumor resistance to radiation therapy [100]. *Nrf2 *deficient mice exposed to radiation had a reduced lifespan compared to *Nrf2* WT mice further supporting a role for NRF2 in radiation treatment [101]. Therapeutic blockade of NRF2 and target ARE genes are currently under investigation as a way to enhance the effects of radiation therapy [102]. Lee et al. screened 8000 synthetic compounds to identify small molecules that inhibit antioxidant responses and increase apoptotic death after radiotherapy [102]. 4-(2-Cyclohexylethoxy)aniline (IM3829) inhibited the increase in NRF2-binding activity, and in combination with radiation, significantly inhibited clonogenic survival of human lung cancer cells [102]. In mice with lung cancer xenografts, IM3829/radiation combination inhibited tumor growth more effectively than in control mice [102]. Brusatol, a quasinoid compound identified from the *Brucea javanica* shrub, has been found to specifically inhibit the NRF2 pathway by enhancing the ubiquitination and degradation of NRF2 [103]. The combination of Brusatol with the chemotherapy drug cisplatin reduced cell number and colony formation on A549 lung cancer cells and reduced tumor sizes on nude mice with A549 xenografts [103]. Continued research has indicated that the effectiveness of combination chemopreventive drug therapies may be linked to their ability to facilitate functional metabolite changes in the tumors, for example, decreased expression of transcription factors such as NRF2 [61, 104]. 

The previously mentioned studies demonstrate the dilemma involved in utilizing the NRF2 pathway: beneficial in suppression of carcinogenesis or resistant to chemotherapy and oncogenic [13]. The answer may not only depend on the stage of carcinogenesis/tumorgenesis but also utilizing a combined genetic and pharmacological approach. While mouse studies combining these approaches have been reviewed in Sporn and Liby [13], reciprocal studies in humans are not as easy to accomplish.

## 5. Conclusions

Based on the studies reviewed, it appears that both mutations leading to elevated *NRF2* expression or a lack of *NRF2 *are effective in lung cancer treatment, largely determined by the stage of carcinogenesis studied (i.e., early during initiation or promotion compared to metastasis)—two radically diverse findings. In general, most of the human studies reviewed were focused on lung AC, but none assessed stage, morphology differences or nodal involvement, thus a definitive pattern of expression in human tumors was not determined. The human epidemiological studies done in several subtypes, including AC and SCC, demonstrated increased NRF2 expression in tumors which is further supported by several of the studies done in knockout mice. In the mouse studies, Nrf2 has a pro-tumorigenic effect in the earlier stages, likely promotion and progression, whereas during metastasis, Nrf2 is observed to be anti-tumorigenic. Overall, the mechanism by which these pro-tumorigenic effects occur likely involves increased cytoprotection, including decreased cell death (by apoptosis and necrosis) and increased proliferation, increased detoxification and upregulation of antioxidant pathways regulated by oncogenes, such as Kras, commonly mutated during initiation events in humans and mice [37, 74]. This ultimately produces a more favorable pulmonary microenvironment for the tumors to develop [77]. In the case of the LLC metastasis model, ROS suppression of CD8^+^ T cells leading to suppression of the entire immune system is likely the culpable mechanism [78]. The findings in mice may also explain the differences observed between activating the NRF2 pathway and chemotherapeutic resistance, since not all studies examined every stage of carcinogenesis. Some of the therapies discussed are also not necessarily NRF2-specific, such as sulforaphane [95], and thus, other mechanistic pathways cannot be excluded. Thus, there are more studies that need to be done to fully understand the role of NRF2 in lung cancer, including assessment of the different types and stages of lung cancer. It may be that only certain stages and subtypes will be sensitive to the effects of *NRF2* mutations. The discovery that the NRF2 pathway has a dual role in cancer should not be seen as a death knell for the chemotherapeutic/chemopreventive drugs that utilize this pathway. What is of importance is that the biologic context in which these drugs are administered must be considered to maximize the efficacy of any cancer treatment regimen. As shown from the research already conducted, the chemotherapy options currently available appear to be highly dependent on the homeostatic state of the NRF2 pathway within the actual lung tumor. 
